# Modeling user rating preference behavior to improve the performance of the collaborative filtering based recommender systems

**DOI:** 10.1371/journal.pone.0220129

**Published:** 2019-08-01

**Authors:** Mubbashir Ayub, Mustansar Ali Ghazanfar, Zahid Mehmood, Tanzila Saba, Riad Alharbey, Asmaa Mahdi Munshi, Mayda Abdullateef Alrige

**Affiliations:** 1 Department of Software Engineering, University of Engineering and Technology, Taxila, Pakistan; 2 Department of Computer Engineering, University of Engineering and Technology, Taxila, Pakistan; 3 College of Computer and Information Sciences, Prince Sultan University, Riyadh, Saudi Arabia; 4 College of Computer Science and Engineering, University of Jeddah, Jeddah, Saudi Arabia; 5 Information Systems Department, Faculty of Computing and Information Technology, King Abdulaziz University, Jeddah, Saudi Arabia; Education University of Hong Kong, CHINA

## Abstract

One of the main concerns for online shopping websites is to provide efficient and customized recommendations to a very large number of users based on their preferences. Collaborative filtering (CF) is the most famous type of recommender system method to provide personalized recommendations to users. CF generates recommendations by identifying clusters of similar users or items from the user-item rating matrix. This cluster of similar users or items is generally identified by using some similarity measurement method. Among numerous proposed similarity measure methods by researchers, the Pearson correlation coefficient (PCC) is a commonly used similarity measure method for CF-based recommender systems. The standard PCC suffers some inherent limitations and ignores user rating preference behavior (RPB). Typically, users have different RPB, where some users may give the same rating to various items without liking the items and some users may tend to give average rating albeit liking the items. Traditional similarity measure methods (including PCC) do not consider this rating pattern of users. In this article, we present a novel similarity measure method to consider user RPB while calculating similarity among users. The proposed similarity measure method state user RPB as a function of user average rating value, and variance or standard deviation. The user RPB is then combined with an improved model of standard PCC to form an improved similarity measure method for CF-based recommender systems. The proposed similarity measure is named as improved PCC weighted with RPB (IPWR). The qualitative and quantitative analysis of the IPWR similarity measure method is performed using five state-of-the-art datasets (i.e. Epinions, MovieLens-100K, MovieLens-1M, CiaoDVD, and MovieTweetings). The IPWR similarity measure method performs better than state-of-the-art similarity measure methods in terms of mean absolute error (MAE), root mean square error (RMSE), precision, recall, and F-measure.

## Introduction

The advancements in machine learning revolutionized the e-commerce business in the last decade. The companies are taking advantage of these advancements by providing users a plethora of online resources for shopping. People are now more interested in buying things online rather than in the old traditional way. In addition, users connect with their friends and colleagues through social networking sites and get reviews about different products. This paradigm shift of shopping and user attitude led to the information overload problem, where users can buy items from millions of online shopping stores. This leads companies to deal with so-called big data problem [1]. Recommender systems aim at solving the information overload problem by recommending products and information to users based on their need and preferences of the community [2, 3].

A few renowned examples of recommender system are Amazon [4], YouTube [5], and Google news [6]. Recommender system uses several categories for creation and generation of information [7]. There are two basic entities in the recommender systems, namely users and items. An active user is a user that is utilizing the recommender system, expresses opinions and provides ratings about different products. The recommender systems apply intelligent filtering methods to rate and recommend items to active users. The two main categories of recommender system are content-based filtering (CBF) and collaborative filtering (CF). The CBF gathers information using the content of items. The main concept of CBF is that the users may like a similar type of items as they liked in the past. In this type of recommender system filtering, user profile record the user interaction with the recommender system and preserve the users’ interests and preferences. For example, LIBRA which is a recommender system for the books. The main concept of CF is that people who agree in the past also agree in the future too. An active user makes a prediction on the target items to find out other similar users called neighbors. The CF is further divided into two types, which are memory-based CF and model-based CF. The memory-based CF uses the rating data to find similar users or items [8]. Different commercial systems are using the memory-based CF for the recommendation. It is efficient and easy to implement. The main focus of this article is to overcome the issues of memory-based CF for the recommender system. The model-based CF discovers latent factors and uses this latent factor to make predictions. Some common examples of the model-based CF are clustering models [9], Bayesian networks[10], singular value decomposition (SVD)[7], and kernel-mapping recommender (KMR) system based algorithms[11]. The memory-based CF gives more significant results for the recommender system. However, its shortcoming is that it takes too much time in case of a large number of users and items. The model-based CF gives faster recommendations as compared to the memory-based CF because it can run off-line to reduce recommendation time. However, shortcomings of model-based CF are that it cannot gives accurate results as compared to the memory-based CF as well as it cannot give out of box recommendation. Despite several benefits, CF also suffers from some limitations like data sparsity and cold-start problems [7]. Data sparsity problem arises when the number of rated items are very low as compared to the items to predict. It occurs due to an overlap between the items rated by two users is too narrow or not exist. The cold start scenario is a problem for a particular item, which holds no ratings or an extremely low number of ratings [12]. In some conditions, CF lacks the ability to provide reliable recommendations due to diverse user interests or when items have different contents [7]. In E-commerce, this problem is called Multiple-interest and Multiple-content recommendation problem [13]. Considering the aforementioned problems of CF, researchers proposed different hybrid methods to improve the performance of the recommender systems such as demographic and semantic information [14–19]. These recommender system methods also use traditional similarity measures like the Pearson correlation coefficient (PCC) [13, 20, 21].

Traditional CF-based methods compute similarities between users for all co-rated items as well as for those items that are different than the target items. Therefore, the neighbors for the active user remain the same even for different items. The most important part of the CF-based method is to find similarities between different users. The commonly used recommender systems are based on traditional similarity measures like PCC or cosine vector similarity [13, 20, 21], which consider only local context information. There are still some issues in traditional similarity measures despite their enormous success. Traditional similarity measures calculate user similarity without considering user RPB. Generally, many users do not rate items according to their quality. These users can be categorized into two types; one that rates every item with almost the same rating leading to zero variance in their ratings. Second, those who give an average rating to all items regardless of the item quality. This creates a serious problem while calculating similarities among users, which often leads to poor recommendations[22]. The miniature research is carried to handle this type of user behavior despite its high impact on recommendations [23–26]. There are several design objectives, which need to be intended to make the recommender system successful, which are discussed below:

**a) Accurate:** Accuracy is one of the most important design objectives of the recommender system. The accuracy helps to build the trust of users when they interact with the recommender system. When a user buys any product recommended to him and starts using it after some time the user realize that the system has given the wrong recommendation about that product. Consequently, a user stops trusting that recommender system. Therefore, the main objective of a recommender system is to give an accurate prediction for items to any user. The proposed IPWR similarity measure method gives accurate recommendations as compared with the state-of-the-art similarity measure methods used for the recommender systems.

**b) Scalable:** A good recommender system should be able to handle large datasets and generate predictions in real time. When the number of users and items increases, the search space grows as well, then it may be difficult to give result in real time, if the recommender system is not scalable. There is always a conflict between accuracy and scalability of a recommender system.

**c) Overspecialization:** In CF-based methods, items are recommended to a user, which are most similar to a user profile. Typical methods of recommender systems cannot give any recommendations about non-co-rated items. The IPWR similarity measure method also overcomes such a scenario by considering ratings of the non-co-rated items.

In this article, we present a novel method for recommender system known as IPWR similarity measure. It takes into account the user RPB towards an item rating to improve standard PCC similarity measure method. To record the user RPB, two methods are proposed: the first method uses mean and variance for each user and it is known as IPWR with variance. The second method uses mean and standard deviation (SD) for each user and it is known as IPWR with SD. The results from either method are then linearly combined with improved PCC similarity measure method. The performance of the IPWR similarity measure method is evaluated on the four state-of-the-art datasets for recommender systems using state-of-the-art similarity measure methods. The IPWR similarity measure outperforms state-of-the-art similarity measures in terms of MAE, RMSE, precision, recall, and F-measure.

The main contributions of this article are as follows:

1. A simple yet highly effective similarity measure method is proposed to model the rating preference behavior (RPB) of users.
2. Standard PCC similarity is improved by overcoming some of its shortcomings. These shortcomings are discussed in a subsection of “Related Work” section entitled as “Shortcomings of standard PCC”.
3. Improved PCC similarity is then adaptively combined with RPB of users.
4. The IPWR similarity measure method not only considers local context information but also take into account the global preference of user ratings.
5. The IPWR similarity measure method can handle the scenarios if no co-ratings are found between two users, as in generally cold start scenarios.

The rest of the sections of this article are organized as follows: Section 2 describes the state-of-the-art literature related to recommender systems. Section 3 describes the methodology of the IPWR similarity measure. Section 4 presents the experimental results and discussion of the IPWR similarity measure method and its comparison with state-of-art similarity measure methods. Section 5 concludes the IPWR similarity measure method and presents future research directions.

## Related work

Collaborative filtering (CF) is now commonly used in many fields for personalized recommendation [2, 12, 13, 27–32]. However, there are also some issues in collaborative filtering (CF), like accuracy, scalability, and cold start, etc. In this paper, the main focus is to improve the prediction accuracy. In CF, items are recommended to users’ according to their preferences, therefore, it is very important that the history of users’ preferences must be available. Different researchers worked on prediction accuracy to improve the performance of the recommender systems. For instance, Ahn et al. [20] propose a solution for CF known as proximity impact popularity (PIP) measure to address the shortcomings of standard PCC and cosine similarity. The PIP measure is the combination of three different aspects of user ratings, which are proximity, impact, and popularity. The PIP similarity only considers the local information of user rating, while the global preference of user ratings is ignored. Moreover, the results of the recommender system using PIP similarity measure are not normalized, which makes it difficult to combine it with other similarity measures. To resolve this issue, the weighted Pearson correlation coefficient (WPCC) method is proposed in. In WPCC[33], the idea of detaining confidence is considered that can be placed on the neighbors. When the number of rated items increases, the confidence also increases and vice versa. Jamali et al.[34] propose a similarity measure, which is based on the sigmoid function. This similarity measure can weaken the similarity of small common rated items among users. J. Bobadilla[35] propose adjusted cosine similarity to overcome the deficiencies of traditional cosine similarity, however, it does not consider the users’ preferences.

Bobadilla et al.[36] propose a novel similarity measure, which utilizes two similarity measures that are the mean squared difference and Jaccard similarity measure. Another metric called mean Jaccard difference (MJD) is proposed to address the cold start problem. Three steps are included in this metric to address the cold start problem. Firstly, the similarity metric is selected. The second step is an evaluation, in which weights are evaluated using neural networks. The last step is a prediction, which is obtained according to the selected similarity metric. [35]proposed a novel similarity measure known as a singularity-based similarity measure. In this similarity measure, it is assumed that the obtained results can be improved by taking contextual information. The user ratings are grouped as positive and negative and the singularity value of user and item is computed. The experimental results show the effectiveness of the proposed similarity. The significance-based similarity measure is proposed by Bobadilla et al.[35]. In this method, the significance of an item, the significance of a user, and the significance of an item for a user are computed. Then according to significance, similarity among users is computed using a standard Pearson correlation coefficient or cosine similarity. It also uses a data smoothing technique for similarity measure, which is the most widely used technique of recommender systems. Different sparsity measures are also used to improve the accuracy of the recommender system. H. Ma et al. [37] propose a similarity measure, in which information of users and items is taken into account and threshold for both are set, respectively. SongJie Gong et al.[38] propose another method to fill the missing ratings by merging SVD and item-based recommender. It uses the item-based method to recommend items to the user.

Szwabe et al. [39] propose a hybrid recommender system method that occupies two-stage data. It processes the data with content features that describe the items and users’ preferences. It improves the accuracy of a system without raising the computational complexity. Moreover, probabilistic matrix factorization is also merged in the recommender system to address issues like data sparsity, cold start, etc. N. Polatidis[40] also propose a novel similarity measure, which uses four different thresholds on a number of co-rated items using PCC to improve the accuracy of the recommender system. Liu et al.[33] also propose a novel similarity measure known as the new heuristic similarity method (NHSM). It computes three parameters, which are proximity, significance, and singularity for each co-rated item. After that, each computed parameter is multiplied by modified Jaccard similarity. The obtained similarity is then again multiplied with a function named as URP to obtain the resultant NHSM similarity [20]. The computation of NHSM-based similarity is complex and lengthy, which makes it difficult to produce a result in real time for the recommender system. All factors in NHSM are again and again multiplied, which ultimately weakens the performance and combining these results with some other similarity measure becomes difficult. A novel similarity measure based on the Bhattacharyya coefficient is proposed by Bidyut Kr. Patra et al. [41]. This method considers both co-rated and non-co-rated items for similarity measure. The resultant similarity measure is a linear combination of the Bhattacharyya coefficient, PCC similarity, and Jaccard similarity. Shuang-Bo Sun et al. [42] propose a novel similarity measure, which combines triangle and Jaccard similarities to improve the performance of the recommender system. Sadasivam et al. [43] propose a novel similarity measure for recommender system, which modifies the Bhattacharyya coefficient using an exponential function and then combined it with Jaccard followed by proximity, significance, and singularity (PSS) measures using a weighted scheme.

### Shortcomings of standard PCC

The standard PCC suffers from some shortcomings, which are discussed below in conjunction with Cosine and CPCC similarity measures.

#### Shortcoming 1: Flat value of ratings

In case user1 rating vector is flat such as (1,1,1) or (3,3,3) or (5,5,5) and user2 rating vector is (1,5,1), PCC will be not a number (NaN). Cosine value will be 0.777 and CPCC value depends upon whether the rating vector is above, equal to or below the median rating value of the rating scale. CPCC value will be +0.333, if rating vector consists of rating values less than median value (i.e. median value = 3), and will be -0.333 if rating values are greater than median value and CPCC, is NaN if all rating values are 3.

#### Shortcoming 2: Only single co-rated item

In case two users contain a single co-rated item, then PCC will be NaN and Cosine will be 1.0. There are two cases for CPCC. In the first case, when the value of rating for both users is equal then the value of CPCC is 1.0 for all values above or below than median value and NaN if rating value is equal to the median value. In the second case, if both users common rating value is different, then CPCC is also NaN.

#### Shortcoming 3: Ignorance of user rating preference behavior (RPB)

The rating preferences may vary from user to user. Some users may rate every item high and some may rate every item low. This scenario of user RPB is not considered in standard PCC.

#### Shortcoming 4: Ignorance of corresponding item average rating in case of user-based CF

In case of user-based CF, standard PCC only consider the average rating of users and ignores the average rating of the corresponding item. Similarly, in the case of item-based CF, user averages are also ignored.

Keeping in view the aforementioned shortcomings of the standard PCC, the proposed IPWR similarity measure method is named as improved PCC weighted with RPB (IPWR). In the IPWR similarity measure method, user RPB is modeled as a Cosine function of user averages and variance or SD. Almost all the aforementioned method in the related work, as well as state-of-the-art similarity measure methods for recommender system, ignore this behavior of a user rating. After that, calculated user RPB is linearly combined with improved PCC to enhance the performance of the IPWR similarity measure method.

## The IPWR similarity measure method

In this section, we explain the methodology of the IPWR similarity measure that is used in memory-based CF to improve the performance of the recommender system. We denote the set of users by *U* = {*a*,*b*,*c*,…,*z*}3320 and a set of items are denoted by *I* = {*i*_0_,*i*_1_,*i*_2_,…,*i*_*m*_}. Each user (e.g. denoted by *a*) rates a set of items denoted by *I*_*a*_. The rating of a user *a* for item *i* is denoted by *R*_*a*,*i*_ and it can be any real number (normally ratings are represented by real numbers in some range [min, max]). The mathematical representation of different similarity measures that are used as a performance comparison with the proposed IPWR similarity measure is presented in Table 1.

**Table 1:** A mathematical representation of different similarity measures.

Similarity measure	Mathematical form
PCC[40]	$$Sim\_PCC(a,b)=\frac{\Sigma_{j\in Ia\cap Ib}(Ra,j-\bar{R}a)(Rb,j-\bar{R}b)}{\sqrt{{\Sigma_{j\in Ia}(Ra,j-\bar{R}a)}^{2}}\sqrt{{\Sigma_{j\in Ib}(Rb,j-\bar{R}b)}^{2}}}$$ (1) where *j* is the set of common rated items between user *a* and *b*. *Ra*,*j* is the rating of user *a* for an item *j* and ${\bar{R}}_{a}$ is the average rating of user *a*.
CPCC [43]	$Sim\_CPCC(a,b)=\frac{\Sigma_{j\in Ia\cap Ib}(Ra,j-R_{med})(Rb,j-R_{med})}{\sqrt{{\Sigma_{j\in Ia}(Ra,j-R_{med})}^{2}}\sqrt{{\Sigma_{j\in Ib}(Rb,j-R_{med})}^{2}}}$ (2) where *j* is the set of common rated items between user *a* and *b*. *R*_*a*,*j*_ is the rating of user *a* for an item *j* and *R*_*med*_ is the median rating on the rating scale.
WPCC[33]	$$Sim\_WPCC(a,b)=\left\{ \begin{array}{l} Sim\_PCC(a,b).\frac{\left|j\right|}{H},|j|\leq H\\ Sim\_PCC(a,b),Otherwise \end{array}\right.$$ (3) where *H* is an experimental value and is set to 50 in[33].
SPCC [42]	$Sim\_SPCC(a,b)=Sim\_PCC(a,b).\frac{1}{1+{exp}^{\left(-\frac{\left|j\right|}{2}\right)}}$ (4)
PIP [20]	$Sim\_PIP(a,b)=\sum_{j\in Ia\cap Ib}PIP(R_{a,j},R_{b,j})$ (5) where *PIP* similarity measure basically consists of three factors, proximity, impact, and popularity.
NHSM[33]	$Sim\_PSS(a,b)=\sum_{j\in Ia\cap Ib}PSS(R_{a,j},R_{b,j})$ (6) where *PSS* basically consists of three factors, proximity, significance, and singularity. These three factors are then combined with modified Jaccard similarity and URP.

As discussed earlier, almost all methods of the similarity measure for the recommender system uses the co-ratings provided by the user. There are many users whose rating preference behavior is different than normal users. They tend to rate items according to their own behavior. Some users may rate every item low whether the item is good or bad. They may do this for bad items or even for good items. There is the second category of users who rate every item high whether that item is good or bad. These types of behavior of the user are termed as rating preference behavior (RPB). In this article, to handle such behaviors, the IPWR similarity measure method uses variance or standard deviation (SD) of each user using a Cosine function. The variance for the user *a* is calculated as follows:

$${var}_{a}=\frac{\Sigma_{j\in I_{a}}(R_{a,j}-{\bar{R_{a}})}^{2}}{\left|I_{a}\right|}$$ (7)

where *R*_*a*,*j*_ represent the rating of user *a* for item *j*, ${\bar{R}}_{a}$ represents the mean of ratings for the user *a* and *I*_*a*_ represents a set of items rated by user *a*. The SD for the user *a* can be calculated as follows:

$${SD}_{a}=\sqrt{{var}_{a}}$$ (8)

where *var*_*a*_ in Eq (8) denotes the variance of user*a*. The calculated values of variance and SD can be used to calculate RPB for two users separately. The *RPB*_(*a*,*b*)_ function which uses variance is denoted by *RPB*_(*a*,*b*)_*using var* and if *SD* is used then it is denoted by *RPB*_(*a*,*b*)_*using SD* and mathematical represented using Eq (9) and Eq (10), respectively as follows:

$${RPB}_{\left(a,b\right)}using\;var=cos(|{\bar{R}}_{a}-{\bar{R}}_{b}|.|{var}_{a}-{var}_{b}|)$$ (9)

$${RPB}_{\left(a,b\right)}using\;SD=cos(|{\bar{R}}_{a}-{\bar{R}}_{b}|.|{SD}_{a}-{SD}_{b}|)$$ (10)

In Eq (9) and Eq (10), ${\bar{R}}_{a}$ and ${\bar{R}}_{b}$ represents the mean of ratings by user *a* and mean of ratings for user *b*, respectively. The cosine function is used to model the RPB of two users. The cosine function is the most commonly used function, which is used by the similarity measure methods either in CF or CB-based recommender systems in the literature. In addition, if two users have the same average rating and variance or SD value, then there RPB will be equal to 1. The use of the Cosine function also results in normalized values whose range is from -1 to +1, which is another reason to use cosine function. In the proposed IPWR similarity measure method, we intend to improve the standard PCC. The standard PCC works only on co-rated items and suffers from shortcoming 4 as discussed in the subsection of related work section entitled as “shortcomings of standard PCC”. To tackle shortcoming 4, both user and item average ratings are used as mentioned in Eq (11). The resultant similarity is given the name of improved PCC similarity measure which is denoted by *Sim*_*IPCC*. Furthermore, the standard PCC ignores users rating pattern, which is also estimated by IPWR similarity measure method using full rating information in the form of user averages and variance or SD.

$${Sim\_IPCC}_{\left(a,b\right)}=\frac{\Sigma_{j\in I_{a}\cap I_{b}}[\left(R_{a,j}*{\bar{R}}_{a})-(R_{a,j}*{\bar{R}}_{j}\right)]*[\left(R_{b,j}*{\bar{R}}_{b})-(R_{b,j}*{\bar{R}}_{j}\right)]}{\sqrt{{\Sigma_{j\in I_{a}}[\left(R_{a,j}*{\bar{R}}_{a})-(R_{a,j}*{\bar{R}}_{j}\right)]}^{2}}\sqrt{{\Sigma_{j\in I_{b}}[\left(R_{b,j}*{\bar{R}}_{b})-(R_{b,j}*{\bar{R}}_{j}\right)]}^{2}}}$$ (11)

where the variables involved in Eq (11) are the same as used in Table 1 for standard PCC. The final similarity is named as improved PCC weighted with *RPB*_(*a*,*b*)_ and denoted by *IPWR*_(*a*,*b*)_ is mathematically represented using Eq (12). The IPWR similarity measure considers both *RPB*_(*a*,*b*)_ and *Sim_IPCC*_*(a*, *b)*_ by combining both factors using an adaptive weighting scheme. Two weights *α* and *β* are chosen, *α* is applied to *RPB*_(*a*,*b*)_ and *β* is applied to *Sim_IPCC*_*(a*, *b)*_. This also ensures that IPWR similarity measure method considers the user rating behavior and it also normalizes high rating effect as well as the low rating effect of each user.

$${IPWR}_{\left(a,b\right)}with\;variance=\alpha .{RPB}_{\left(a,b\right)}using\;var+\beta .{Sim\_IPCC}_{\left(a,b\right)}$$ (12)

$${IPWR}_{\left(a,b\right)}with\;SD=\alpha .{RPB}_{\left(a,b\right)}using\;SD+\beta .{Sim\_IPCC}_{\left(a,b\right)}$$ (13)

The weights of *α* and *β* are determined in a separate subsequent section entitled as “Determining best weights for α and β”. The range of values for *IPWR*_(*a*,*b*)_ are from -1 to +1 and thus a similarity threshold *θ*_*s*_ is also required to be put on the similarity value generated by Eq (12) or Eq (13). The reason for adding both *RPB*_(*a*,*b*)_ and *Sim_IPCC*_(*a*,*b*)_ is that similarity range of *Sim_IPCC*_(*a*,*b*)_ is from -1 to +1, while *RPB*_(*a*,*b*)_ similarity range is also from -1 to +1. Now if two users have a slightly negative *Sim_IPCC*_(*a*,*b*)_ similarity but a high positive value for *RPB*_(*a*,*b*)_ then the overall similarity value for Eq (12) will become greater than zero implying a positive similarity between these two users. However, if *RPB*_(*a*,*b*)_ is not used in conjunction with *Sim_IPCC*_(*a*,*b*)_ then both users are treated as a dis-similar user by *Sim_IPCC*_(*a*,*b*)_. Similarly, if two users have a slightly positive *Sim_IPCC*_(*a*,*b*)_ similarity while a high negative value for *RPB*_(*a*,*b*)_ then overall similarity *IPWR*_(*a*,*b*)_ consider these two users as dis-similar users.

The final recommendations are generated using Eq (14), which is known as Resnick’s formula [44] and either Eq (12) or Eq (13) can be used by *IPWR*_(*a*,*b*)_ similarity measure method and defined mathematically as follows:

$${\hat{R}}_{a,i}={\bar{R}}_{a}+\frac{\Sigma_{b\in NN}{IPWR}_{\left(a,b\right)}.(R_{b,i}-{\bar{R}}_{b})}{\Sigma_{b\in NN}|{IPWR}_{\left(a,b\right)}|}$$ (14)

where *p* denotes a user belonging to nearest neighbor (NN) network of the user *a*. The top similar users of *a* are identified as nearest neighbors of the user *a*.

The pseudo code for the IPWR similarity measure method is outlined below and entitled as “Algorithm 1”. The experimental results of the IPWR similarity measure method are reported using five-fold cross validation[45] on each of the publically available dataset. The Pearson similarity is computed using Eq (11) and RPB is computed using either Eq (9) or Eq (10). The results of step 4 and step 5 are combined using Eq (12). Final prediction is generated using Eq (14).

______________________________________________________________________________________________________

Algorithm 1: Procedure of recommendation by the IPWR similarity measure method

**Input:** Rated dataset

**Output:** Predicted rating $\hat{R}$

______________________________________________________________________________________________________

    1. Perform five-fold cross validation on ratings dataset to obtain test and training set.

    2. Select target user *a* and test item *i*_0_ from the test set.

    3. Find all users *b* who have rated *i*_0_ in the training set.

    4. Find improved Pearson similarity *Sim_IPCC*_(*a*,*b*)_ between *a* and *b* using Eq (11).

    5. Find rating preference behavior *RPB*_(*a*,*b*)_ of *a* and *b* using either Eq (9) or Eq (10).

    6. Combine results from steps 4 and 5 using either Eqs (12) or (13).

    7. Make prediction $\hat{R}$ on target item *i*_0_ of target user *a* using Eq (14).

    8. Return $\hat{R}$.

_____________________________________________________________________________________

Consider an example, which demonstrates the working of the IPWR similarity measure method. In this example, Table 2 is showing an instance of a user-item based rating matrix. In the current situation, the rating matrix consist of five users and five items. The five users are denoted by *a* to *e*, while five items are denoted by *i*_1_ to *i*_5_. In this example, we want to predict a rating of an item *i*_5_ for the user *a*. The distinct feature of this user-item based rating matrix is that user *e* rating vector is flat which corresponds to shortcoming 1 of standard PCC as mentioned earlier in the subsection entitled as “Shortcomings of standard PCC”. For this reason, although user *a* and user *e* consist of exactly the same value of co-rated items, PCC is NaN. User *a* and user *c* consist of single co-rated item (i.e. *i*_3_ only), which corresponds to the shortcoming 2 of the standard PCC.

**Table 2:** An example of a user-item based rating matrix.

	Items				
*Users*	*i*_*1*_	*i*_*2*_	*i*_*3*_	*i*_*4*_	*i*_*5*_
***a***	*3*	*-*	*5*	*4*	?
***b***	*1*	*-*	*-*	*5*	*1*
***c***	*-*	*3*	*4*	*-*	*-*
***d***	*1*	*-*	*1*	*4*	*2*
***e***	*3*	*-*	*-*	*3*	*3*

Table 3 contain various parameters, which are computed from Table 2. The variance of each user is computed using Eq (7), *RPB* of users is computed using Eq (9), *Sim_PCC* value is computed using Eq (1), and *Sim_IPCC* value is computed using Eq (11).

*IPWR*_(*a*,*b*)_ = 0.5*0.073+0.5*0.0563 = 0.0646

*IPWR*_(*a*,*c*)_ = 0.5*0.978+0.5*1.0 = 0.744

*IPWR*_(*a*,*d*)_ = 0.5*(-0.09) +0.5*(-0.079) = -0.084

*IPWR*_(*a*,*e*)_ = 0.5*(0.785) +0.5*(0.707) = 0.372

**Table 3:** Computed values of different statistics from Table 2.

Average rating of users		Average rating of items		The variance of each user		RPB of users		Sim_PCC		Sim_IPCC	
${\bar{R}}_{a}$	4.0	$\bar{i_{1}}$	2.0	*var*_*a*_	0.667						
${\bar{R}}_{b}$	2.34	$\bar{i_{2}}$	3.0	*var*_*b*_	3.55	(a, b)	0.073	(a, b)	0.447	(a, b)	0.0563
${\bar{R}}_{c}$	3.5	$\bar{i_{3}}$	3.34	*var*_*c*_	0.25	(a, c)	0.978	(a, c)	NaN	(a, c)	1.0
${\bar{R}}_{d}$	2.0	$\bar{i_{4}}$	4.0	*var*_*d*_	1.5	(a, d)	-0.09	(a, d)	0.0	(a, d)	-0.079
${\bar{R}}_{e}$	3.0	$\bar{i_{5}}$	2.0	*var*_*e*_	0.0	(a, e)	0.785	(a, e)	NaN	(a, e)	0.707

From Table 3, it can be noted that *Sim*_*PCC*_(*a*,*c*)_ and *Sim*_*PCC*_(*a*,*e*)_ values are *NaN*, but at the same time values of *Sim*_*IPCC* and *IPWR* are real numbers that effectively abolishing shortcomings 1 and 2 of the standard PCC. The value of *Sim*_*PCC*_*a*,*d*_ = 0.0, which is very interesting. The users *b*,*d*, and *e* rated target item ***i***_**5**_. By applying the similarity threshold *θ*_*s*_, a set of nearest neighbors (NN) for the user *a* are identified and *NN* = {*b*,*e*}. The value of user *b* rating for an item *i*_5_ is 1.0 and the value of user *e* rating for an item *i*_5_ is 3.0.

$${\hat{R}}_{a,i_{5}}=\frac{0.0646\times (1.0-2.34)+0.372\times (3.0-3.0)}{0.0646+0.372}=4.0+\frac{-0.085}{0.436}=4.0-0.194=3.8$$

## Experimental setup and performance evaluation metrics

### Datasets

The standard datasets (namely Epinions [46], MovieLens-100K (ML-100K) [47], MovieLens-1M (ML-1M)[48], CiaoDVD[49], and MovieTweetings [50]) are used for the performance evaluation of the IPWR similarity measure method. The details about these datasets are as follows:

**a) Epinions dataset:** The Epinions is an online community website that allows users to review different products and services. Users can also rate the other user’s review on a numerical scale. This dataset contains 664823 ratings on the scale of 1.0 (worst rating) to 5.0 (best rating) with the step size of 1.0. This dataset contains 139738 items that are rated by 40163 users with 99.90% sparsity. The value of mean rating per user is 10.39 with a maximum of 1023 ratings per user. The value of mean rating per item is 4.75 with a maximum of 2026 ratings per item.

The sparsity is calculated as follows:

$$Sparsity=\left(1-\frac{non\;zero\;entries}{all\;possible\;entries}\right)X\;100$$ (15)

**b) MovieLens-100K (ML-100K) dataset:** The ML-100K dataset contains 943 users that rated different movies on a scale of 1.0 (worst rating) to 5.0 (best rating). The most rated value of this dataset is 4.0. This dataset includes 100000 user ratings over 1682 movies and each user rated at least 20 movies. This dataset is used by different state-of-the-art similarity measure methods for recommender system and its sparsity is 93.70%.

**c) MovieLens-1M (ML-1M) dataset:** The group lens research group collected and made publically available this dataset from the MovieLens website. On this web site, users can rate and review different movies. This dataset contains 6040 users, 3952 movies, and 1000209 user ratings. The ratings take values from 1.0 (worst rating) to 5.0 (best rating) with the step size of 1.0. The sparsity of this dataset is 95.80%. The value of mean rating per user is 15.63 with a maximum of 2314 ratings per user and value of mean rating per item is 269.80 with a maximum of 3428 ratings per user.

**d)CiaoDVD dataset:** The CiaoDVD dataset contains 72665 user ratings on the scale of 1.0 (worst rating) to 5.0 (best rating) with a step size of 1.0. This dataset contains 16121 items rated by 17615 users with 99.90% sparsity. The value of mean rating per user is 1.13 with a maximum of 1106 ratings per user. The value of mean rating per item is 4.48 with a maximum of 424 ratings per item.

**Fig 1:**
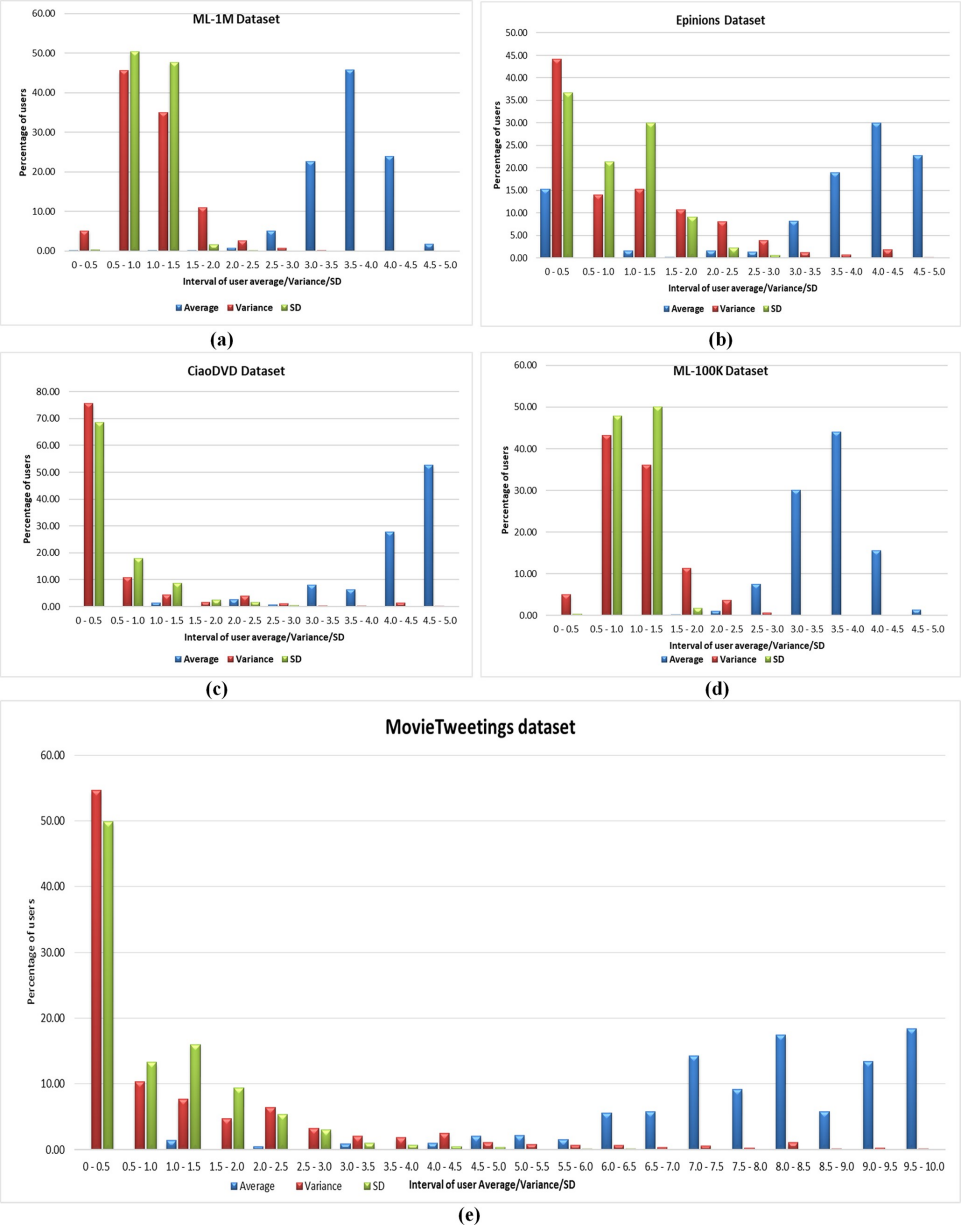
(a-e) Percentage of user average/variance/SD ratings for the reported datasets.

The rating distribution for all four datasets is shown in Table 4. In the IPWR similarity measure method, RPB function comprises of user average rating and variance or SD, so the IPWR similarity measure method considers statistics of user average ratings, variance, and SD in intervals of 0.5. These statistics are shown in (Fig 1A–1E). It can be observed that maximum value of the variance/SD ratings occurs in the interval of 0.5–1.0 and a maximum value of average ratings of the users occur in the interval of 3.5–4.0 for the ML-1M dataset. For the Epinions dataset, the variance/SD ratings of a maximum number of users occur in the interval 0–0.5 while the maximum value of average ratings of the users occurs in the interval of 4.0–4.5. For CiaoDVD dataset, the variance/SD ratings of a maximum number of users also occur in the interval of 0–0.5 and maximum average ratings of the users occur in the interval of 4.5–5.0. Similarly, for ML-100K dataset, the variance/SD ratings of a maximum number of users occur in the intervals of 0.5–1.0/1.0–1.5, while the maximum value of average ratings of the users occurs in the interval of 3.5–4.0. According to the details shown in Fig 1(E) for the MovieTweetings dataset, it can be noted that the variance/SD of more than 50% of users occurs in the interval of 0–0.5. Furthermore, maximum intervals for user average values are found to be 8.0–8.5 and 9.5–10.0. Keeping these facts into view, the RPB function of Eq (9) and Eq (10) give better performance for the Epinions, CiaoDVD, and MovieTweetings datasets as compared to the ML-1M and ML-100K datasets. Its reason is that variance/SD ratings of a maximum number of users occur in the interval of 0–0.5. It is also obvious from the results of (Fig 1A–1E) that user averages and variances/SD ratings are not well distributed. The variance/SD occur in the interval of initial scale to median scale, while maximum average ratings of the users occur in the opposite side (i.e. median scale to maximum scale). This is the main reason behind the modeling of RPB function in terms of user average rating and variance/SD value.

**Table 4:** Rating distributions of the reported datasets.

Rating scale	Ratings of users for each dataset			
	ML-1M dataset	CiaoDVD dataset	Epinions dataset	ML-100K dataset
1.0	56174	2651	43228	6110
2.0	107557	4685	50678	11370
3.0	261197	10074	75525	27145
4.0	348971	22560	194339	34174
5.0	226310	32695	301053	21201
**Total**	**1000209**	**72665**	**664823**	**100000**

## Performance evaluation metrics

The performance of the IPWR similarity measure method is evaluated using five-fold cross-validation due to its extensive usage in state-of-the-art similarity measure methods for recommender system and average results are reported. An alternate choice to five-fold cross-validation is Leave-one-out method which requires high computational complexity as compared to five-fold cross-validation method[45]. The performance of the IPWR similarity measure method is measured in terms of MAE, RMSE, precision, recall, and F-measure. The MAE calculates the average absolute deviation among the predicted ratings given by the recommender system and true ratings given by the user. The RMSE takes an average of squared error result by giving more weight to higher value errors and less weight to smaller value errors. The mathematical representation of the MAE and RMSE are as follows:

$$MAE=\frac{1}{N}\sum_{i=1}^{N}\left|R_{a,i}-{\hat{R}}_{a,i}\right|$$ (16)

$$RMSE=\sqrt{\frac{1}{N}\sum_{i=1}^{N}{\left|R_{a,i}-{\hat{R}}_{a,i}\right|}^{2}}$$ (17)

where *N* denotes the total number of items for which the prediction process is performed. The main goal of any recommender system is to decrease the MAE and RMSE.

The precision and recall assess the specificity and sensitivity of a recommender system by measuring the frequency of items, respectively. The most suitable way to measure the precision and recall is to predict the top *N* items for known ratings. All the experimental results of the IPWR similarity measure method are reported by setting *N* = 5. The fundamental supposition is the division among relevant and irrelevant items in every user’s dataset. Precision and recall are empirically defined in Table 5 and are mathematically expressed in Eq (18) and Eq (19) as follows:

$$Precision=\frac{N_{ms}}{N_{s}}\times 100\%$$ (18)

$$Recall=\frac{N_{ms}}{N_{m}}\times 100\%$$ (19)

**Table 5:** *Confusion matrix*: *Each row represents an actual class and each column represents predicted class*.

Actual class	Predicted class		Total
	Selected	Not selected	
Relevant	*N*_*ms*_	*N*_*mn*_	*N*_*m*_
Irrelevant	*N*_*cs*_	*N*_*cn*_	*N*_*c*_
	*N*_*s*_	*N*_*n*_	*N*

However, a tradeoff exists for precision and recall in the sense that if one value increases then other value decreases and vice versa. To overcome this tradeoff, the state-of-the-art similarity measure methods for recommender system also uses F-measure as a performance evaluation metric, which is mathematically defined using Eq (20) as follows:

$$F-measure=2\times \frac{Precision\times Recall}{Precision+Recall}$$ (20)

## Experimental results and discussions

Three different cases are used to measure the performance of the IPWR similarity measure method. In the first case, the impact of the varying similarity threshold (denoted by *θ*_*s*_) on the performance of the IPWR similarity measure method is analyzed. In the second case, the best weights of *α* and *β* are determined for each dataset using an adaptive weighting scheme. In the third case, the impact on the performance of the IPWR similarity measure method is analyzed by varying neighbor’s size and its performance comparisons are performed with state-of-the-art similarity measure methods. The experimental details about these three cases are given in the following subsections.

### Methods used for comparison

The performance of the IPWR similarity measure method is compared with state-of-the-art similarity measure methods. These similarity measure methods include PCC, CPCC, WPCC, SPCC, Cosine, PIP, Singularity measure. and NHSM. The detail about these state-of-the-art similarity measure methods is as follows:

#### a) PCC similarity measure

The value of PCC similarity measure method is calculated using Eq (1). The range of PCC value is from -1 to +1. The -1 corresponds to the worst similarity value and +1 corresponds to the best similarity value. For all similarity measure methods, a similarity threshold is also required to be imposed on produced similarity values. The value of the similarity threshold (denoted by *θ*_*s*_) for PCC similarity measure is greater than zero. This implies that users having negative similarity are ignored.

#### b) CPCC similarity measure

This similarity measure method is calculated using Eq (2). The CPCC similarity measure categorizes all rating values as positive or negative. A rating value is positive if it is above the median rating of the rating scale and negative if it is below the median rating of the rating scale. For all reported datasets, the value of the median rating is set to 3. Like PCC, CPCC result range is also from -1 to +1 and the similarity threshold (*θ*_*s*_) is also set to greater than zero.

#### c) WPCC similarity measure

This similarity measure method is calculated using Eq (3). This method gives more weight to users whose number of common rated items are greater than some threshold and its value is set to 50 [20]. The range of values for the WPCC similarity measure method is from -1 to +1.

#### d) SPCC similarity measure

This is an exponential version of the standard PCC similarity measure method and it is calculated using Eq (4). Its possible values are from -1 to +1 and the similarity threshold (*θ*_*s*_) is set to greater than zero.

#### e) COSINE similarity measure

This similarity measure method is introduced by [12]. Its possible values are from 0 to 1, which indicate that all users with similarity greater than zero are selected for the prediction process.

#### f) PIP similarity measure

This similarity measure method first computes a Boolean function followed by an agreement between two user ratings. After that, it calculates PIP factors based on whether the agreement is true or false. The value of the PIP similarity measure method is greater than zero and it can be any real number. The value of the PIP similarity measure method is calculated using Eq (5).

#### g) Singularity measure

In this similarity measure, the user ratings are grouped as positive and negative, and the singularity value of user and item is computed. The prediction is generated based upon computed singularity value.

#### h) NHSM similarity measure

This similarity measure method is calculated using Eq (6). This method considers both local and global preference of a user rating. Its value range is from 0 to 1.

### Effect of the similarity threshold

**Fig 2:**
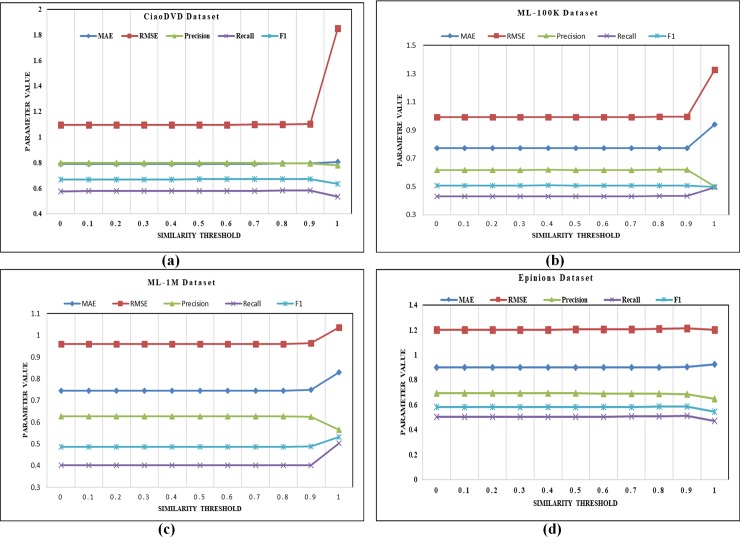
(a-d) Effect of similarity threshold on reported datasets.

To estimate the impact of the similarity threshold (*θ*_*s*_), its values are varied from 0 to 1.0 with a step size of 0.1 at a fixed nearest neighbor size of 5. It is obvious from Fig 2(A) of the CiaoDVD dataset that no significant change is observed until *θ*_*s*_ = 0.9. The worst results for RMSE are produced when *θ*_*s*_ = 1.0 and suddenly jumps from 1.102 to 1.85 when corresponding MAE results remain unchanged. The precision value decreases from 0.794 to 0.779. Similarly, the value of recall at *θ*_*s*_ = 1.0 decreases to 0.533 from 0.582. The value of F-measure reaches to 0.634 from 0.672. Similarly, in Fig 2(B) of the ML-100K dataset, results of all evaluation parameters remain almost the same till *θ*_*s*_ = 0.9. At *θ*_*s*_ = 1.0, MAE value increases from 0.773 to 0.939, RMSE value increases from 0.995 to 1.326. The value of precision at *θ*_*s*_ = 1.0 decreases from 0.619 to 0.499, recall increases from 0.432 to 0.495, and value of F-measure decreases from 0.507 to 0.497. In Fig 2(C) of the ML-1M dataset, similar behavior of ML-100K dataset is observed. After *θ*_*s*_ = 0.9, MAE, and RMSE values are increases, while the value of the precision, recall, and F-measure is decreasing. In Fig 2(D) of the Epinions dataset, MAE values remain almost constant till *θ*_*s*_ = 0.7 and after that, it starts increasing. For RMSE, performance remains almost constant till *θ*_*s*_ = 0.7, and at *θ*_*s*_ = 0.8 and *θ*_*s*_ = 0.9 its increases, while at threshold = 1.0, performance decreases to 1.199. Which means that values of the precision, recall, and F-measure are also decreasing due to an increase in the threshold value. After observing these experimental details of the reported datasets, it can be concluded that values of the MAE and RMSE increases with increase in the value of the similarity threshold (*θ*_*s*_) while values of the precision, recall, and F-measure are decreases.

### Determining best weights for α and β

In order to determine the best weights of *α* and *β* to achieve improved performance of the IPWR similarity measure method as compared with state-of-the-art similarity measure methods, its performance is evaluated by varying different weights of *α* and *β* from 0 to 1. The experimental details about the performance of the IPWR similarity measure method on different weights of *α* and *β* are given in Table 6 for all the reported datasets. In Table 6, the bold values indicate the best weights of *α* and *β* which gives the best performance of the IPWR similarity measure method in terms of the performance evaluation metrics for all the reported datasets. For ML-100K dataset, the best results are found when *α =* 0.5 and *β =* 0.5. Similarly, by setting *α* = 0.1 and *β = 0*.*9*, better results are gathered as compared to the case, when *α* = 0 and *β =* 1.0. This implies that RPB produces an important effect on the recommendation performance of the IPWR similarity measure method. For Epinions, CiaoDVD, and ML-1M datasets, the best performance of the IPWR similarity measure method is obtained by setting weights of *α =* 0.4 and *β =* 0.6. In these datasets, it is also obvious that performance is improved when weights of *α* and *β* are increased from *0*.*0*, 1.0 to 0.1, and 0.9. Furthermore, the worst performance is obtained, when *α =* 1.0 and *β =* 0.0, which indicates that RPB alone is not able to yield good results. In the case of the MovieTweetings (10-star rating) dataset, the best performance of the IPWR similarity measure method is achieved by setting the weight of *α =* 0.4 and *β =* 0.6.

**Table 6:** Estimating the best weights of α and β for reported datasets (bold values in each row indicate the best performance, while bold values of α and β indicate best weights selected on the bases of lowest MAE and highest F-measure values).

**ML-100K (5-star rating) dataset**											
**Evaluation metrics**	α = 0, β = 1.0	α = 0.1, β = 0.9	α = 0.2, β = 0.8	α = 0.3, β = 0.7	α = 0.4, β = 0.6	**α = 0.5, β = 0.5**	α = 0.6, β = 0.4	α = 0.7, β = 0.3	α = 0.8, β = 0.2	α = 0.9, β = 0.1	α = 1.0, β = 0.0
**MAE**	0.788	0.778	0.776	0.775	0.774	**0.773**	**0.773**	**0.773**	**0.773**	0.774	0.804
**RMSE**	1.010	0.998	0.996	0.994	**0.993**	**0.993**	**0.993**	**0.993**	**0.993**	0.994	1.027
**Precision**	**0.619**	**0.619**	**0.618**	**0.619**	0.617	**0.617**	0.617	0.616	0.615	0.614	0.594
**Recall**	0.417	0.426	0.429	0.430	0.430	**0.431**	**0.431**	0.430	0.430	0.430	0.41
**F-measure**	0.496	0.502	0.505	0.505	0.504	**0.506**	0.505	0.504	0.504	0.504	0.483
**Epinions (5-star rating) dataset**											
**Evaluation metrics**	α = 0, β = 1.0	α = 0.1, β = 0.9	α = 0.2, β = 0.8	α = 0.3, β = 0.7	**α = 0.4, β = 0.6**	α = 0.5, β = 0.5	α = 0.6, β = 0.4	α = 0.7, β = 0.3	α = 0.8, β = 0.2	α = 0.9, β = 0.1	α = 1.0, β = 0.0
**MAE**	0.907	0.901	0.900	**0.899**	**0.899**	0.900	0.905	0.908	0.911	0.915	0.925
**RMSE**	1.209	1.203	1.202	**1.201**	**1.201**	1.204	1.210	1.212	1.216	1.220	1.230
**Precision**	0.691	0.692	0.692	0.692	**0.693**	0.691	0.689	0.687	0.685	0.683	0.680
**Recall**	0.497	0.500	0.500	0.501	**0.502**	0.501	0.500	0.498	0.496	0.494	0.489
**F-measure**	0.578	0.580	0.581	0.581	**0.582**	0.580	0.579	0.577	0.575	0.573	0.569
**CiaoDVD (5-star rating) dataset**											
**Evaluation metrics**	α = 0, β = 1.0	α = 0.1, β = 0.9	α = 0.2, β = 0.8	α = 0.3, β = 0.7	**α = 0.4, β = 0.6**	α = 0.5, β = 0.5	α = 0.6, β = 0.4	α = 0.7, β = 0.3	α = 0.8, β = 0.2	α = 0.9, β = 0.1	α = 1.0, β = 0.0
**MAE**	0.802	0.792	**0.791**	**0.791**	**0.791**	**0.791**	0.795	0.797	0.799	0.801	0.804
**RMSE**	1.104	1.096	1.096	1.096	**1.095**	1.096	1.101	1.102	1.105	1.107	1.111
**Precision**	0.796	**0.797**	**0.797**	**0.797**	**0.797**	**0.797**	0.796	0.795	0.795	0.794	0.794
**Recall**	0.571	0.575	0.576	0.576	**0.577**	0.576	0.576	0.575	0.574	0.572	0.571
**F-measure**	0.665	0.669	0.669	0.669	**0.670**	0.669	0.669	0.668	0.667	0.666	0.665
**ML-1M (5-star rating) dataset**											
**Evaluation metrics**	α = 0, β = 1.0	α = 0.1, β = 0.9	α = 0.2, β = 0.8	α = 0.3, β = 0.7	**α = 0.4, β = 0.6**	α = 0.5, β = 0.5	α = 0.6, β = 0.4	α = 0.7, β = 0.3	α = 0.8, β = 0.2	α = 0.9, β = 0.1	α = 1.0, β = 0.0
**MAE**	0.757	0.747	0.745	**0.744**	**0.744**	**0.744**	**0.744**	**0.744**	**0.744**	0.745	0.785
**RMSE**	0.975	0.963	0.961	0.960	**0.959**	**0.959**	**0.959**	**0.959**	**0.959**	0.960	1.004
**Precision**	0.614	0.614	0.614	0.615	**0.616**	0.615	0.614	0.614	0.611	0.410	0.597
**Recall**	0.348	0.356	0.356	0.356	**0.357**	0.356	0.356	0.356	0.356	0.353	0.321
**F-measure**	0.401	0.407	0.408	0.408	**0.409**	0.408	0.408	0.408	0.407	0.405	0.379
**MovieTweetings (10-star rating) dataset**											
**Evaluation metrics**	α = 0, β = 1.0	α = 0.1, β = 0.9	α = 0.2, β = 0.8	α = 0.3, β = 0.7	**α = 0.4, β = 0.6**	α = 0.5, β = 0.5	α = 0.6, β = 0.4	α = 0.7, β = 0.3	α = 0.8, β = 0.2	α = 0.9, β = 0.1	α = 1.0, β = 0.0
**MAE**	1.212	1.159	1.156	1.154	1.154	1.154	1.156	1.158	1.16	1.164	1.199
**RMSE**	1.7	1.646	1.643	1.642	1.642	1.645	1.648	1.651	1.65	1.66	1.703
**Precision**	0.696	0.699	0.699	0.699	0.699	0.699	0.696	0.694	0.691	0.687	0.672
**Recall**	0.514	0.537	0.539	0.539	0.539	0.539	0.541	0.54	0.54	0.539	0.528
**F-measure**	0.591	0.607	0.608	0.608	0.608	0.608	0.607	0.606	0.605	0.603	0.591

### Effect of the number of neighbors and comparison of state-of-the-art similarity measure methods with IPWR similarity measure method

**Fig 3:**
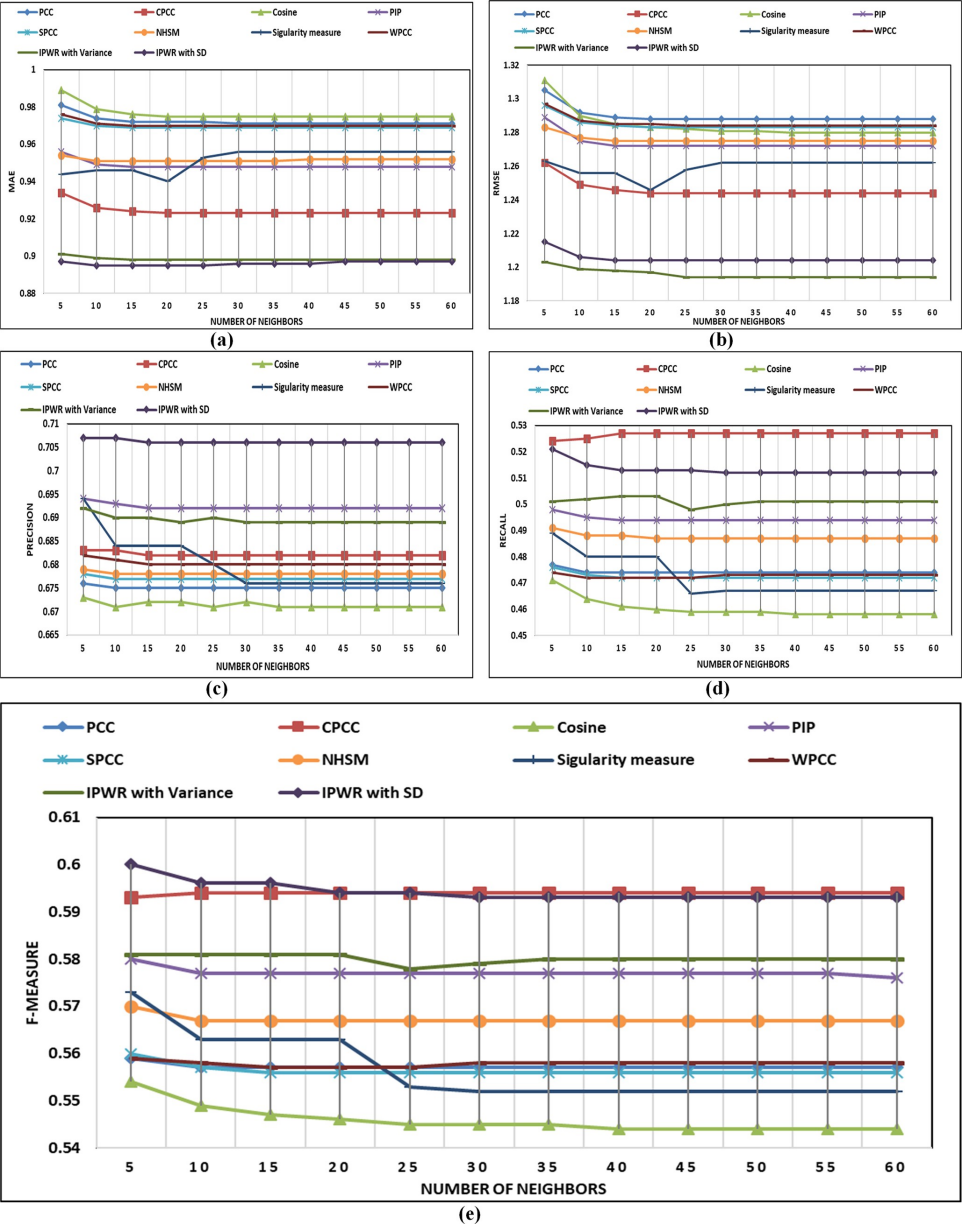
(a-e) Performance comparison of state-of-the-art similarity measure methods with the IPWR similarity measure method in terms of MAE, RMSE, precision, recall, and F-measure on the Epinions dataset.

In this section, details about performance comparison of the IPWR similarity measure method with state-of-the-art similarity measure methods (i.e. Standard PCC, CPCC, WPCC, SPCC, COSINE, PIP, Singularity measure, and NHSM) is carried. The performance of the IPWR similarity measure method and its competitor methods are analyzed in terms of the performance evaluation metrics (i.e. MAE, RMSE, Precision, Recall, and F-measure) by varying a different number of neighbors whose details are shown in (Fig 3A–3E) to (Fig 7A–7E).

**Fig 4:**
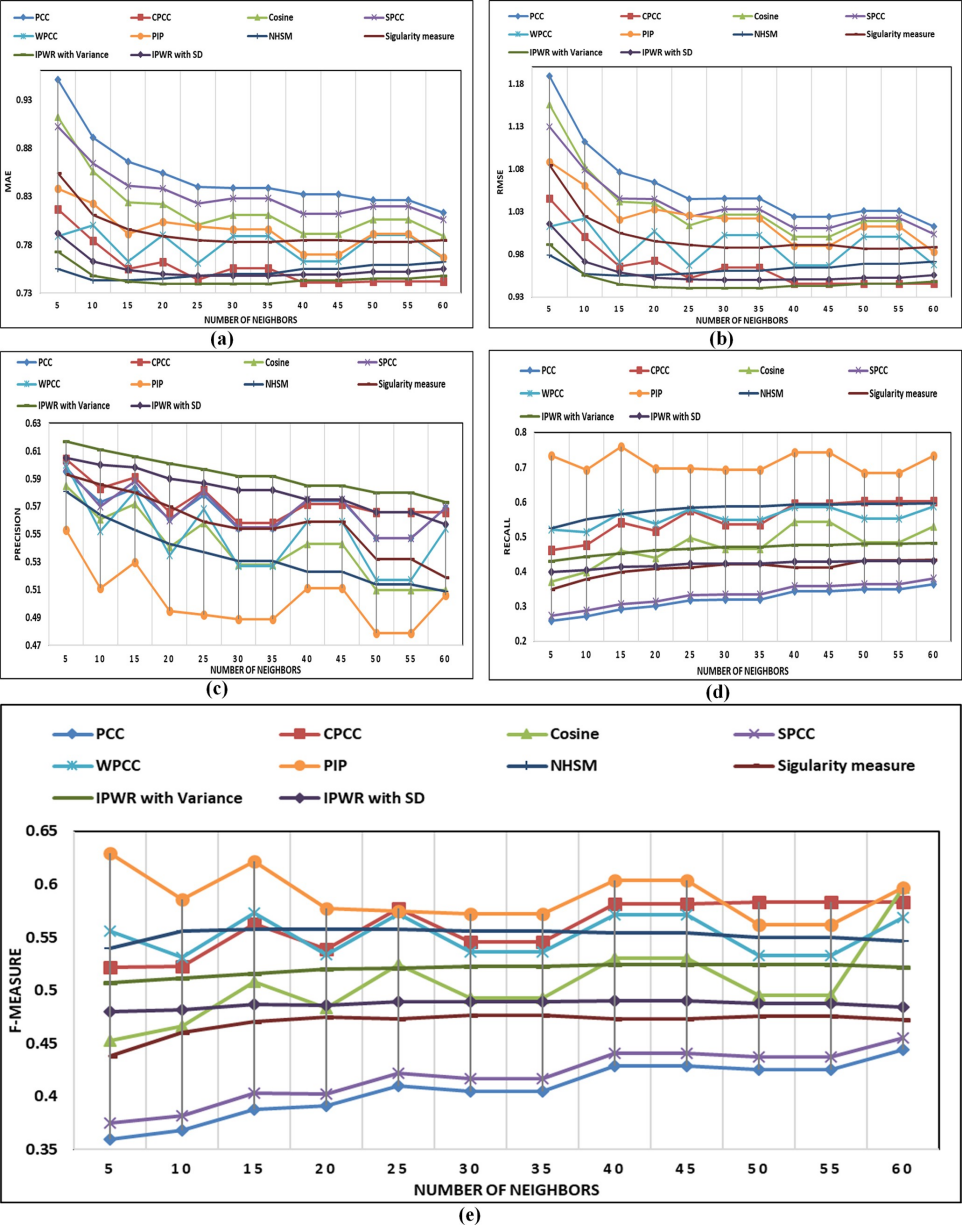
(a-e) Performance comparison of state-of-the-art similarity measure methods with the IPWR similarity measure method in terms of MAE, RMSE, precision, recall, and F-measure on the ML-100K dataset.

**Fig 5:**
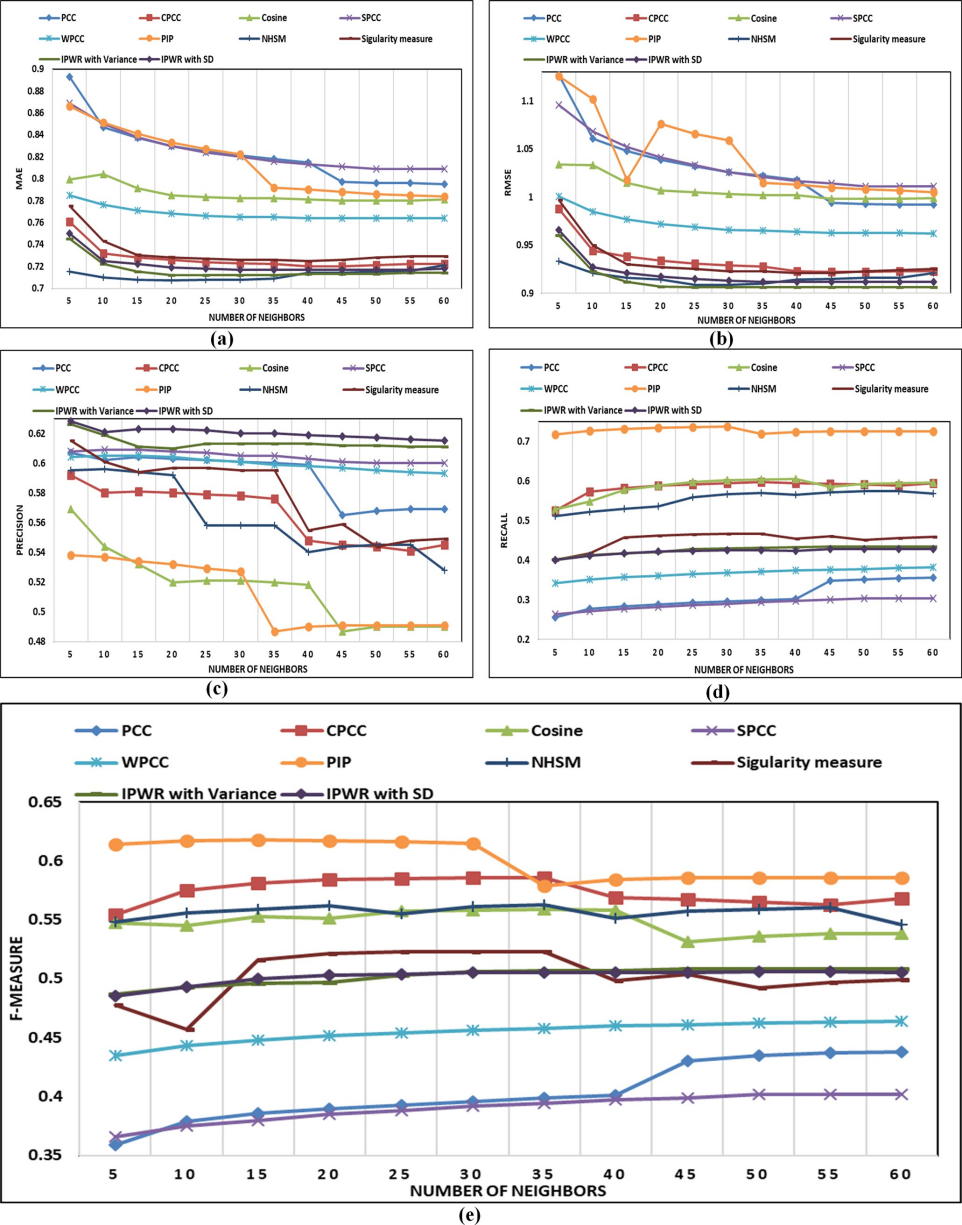
(a-e) The performance comparison of state-of-the-art similarity measure methods with the IPWR similarity measure method in terms of MAE, RMSE, precision, recall, and F-measure on the ML-1M dataset.

**Fig 6:**
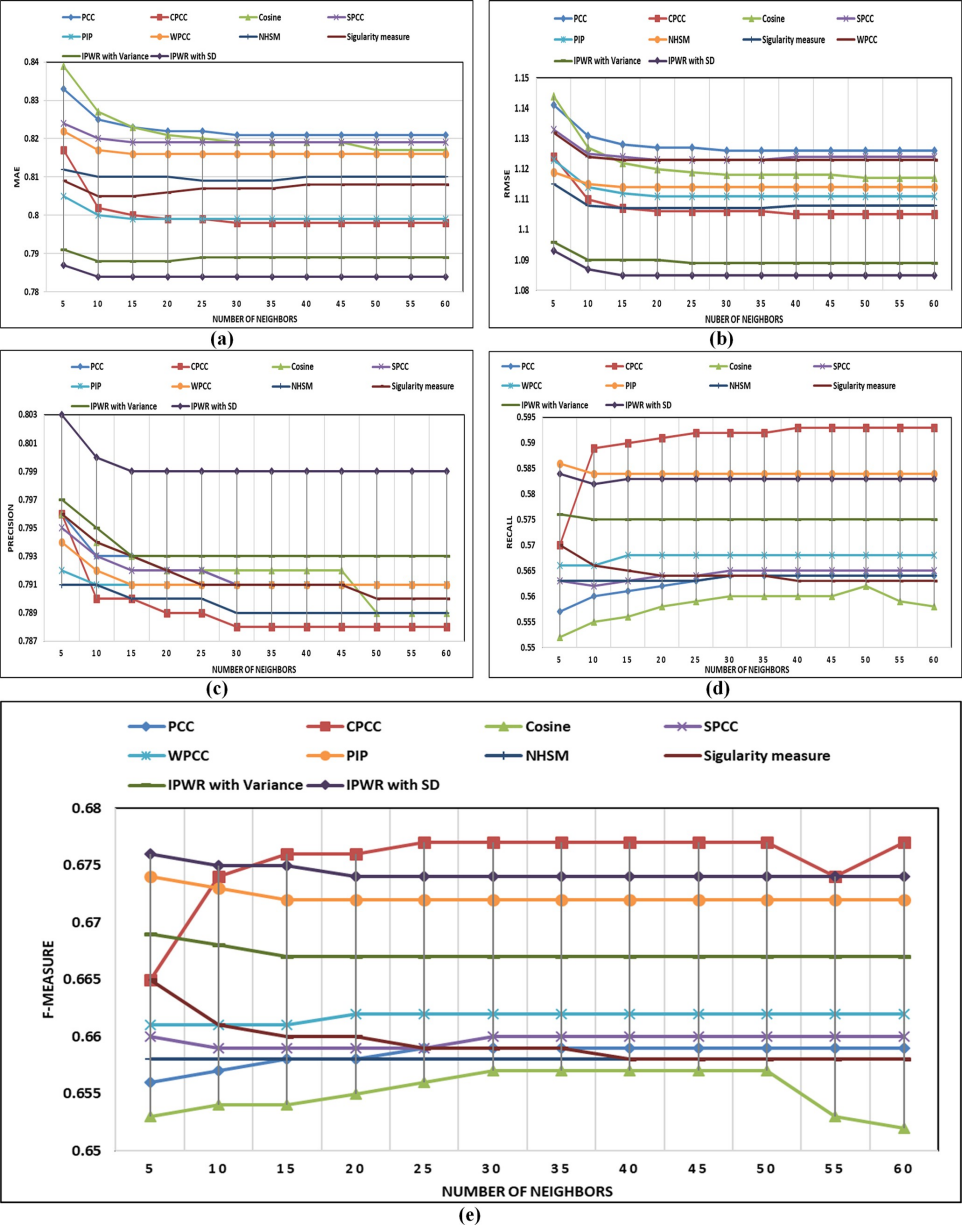
(a-e) The performance comparison of state-of-the-art similarity measure methods with the IPWR similarity measure method in terms of MAE, RMSE, precision, recall, and F-measure on the CiaoDVD dataset.

**Fig 7:**
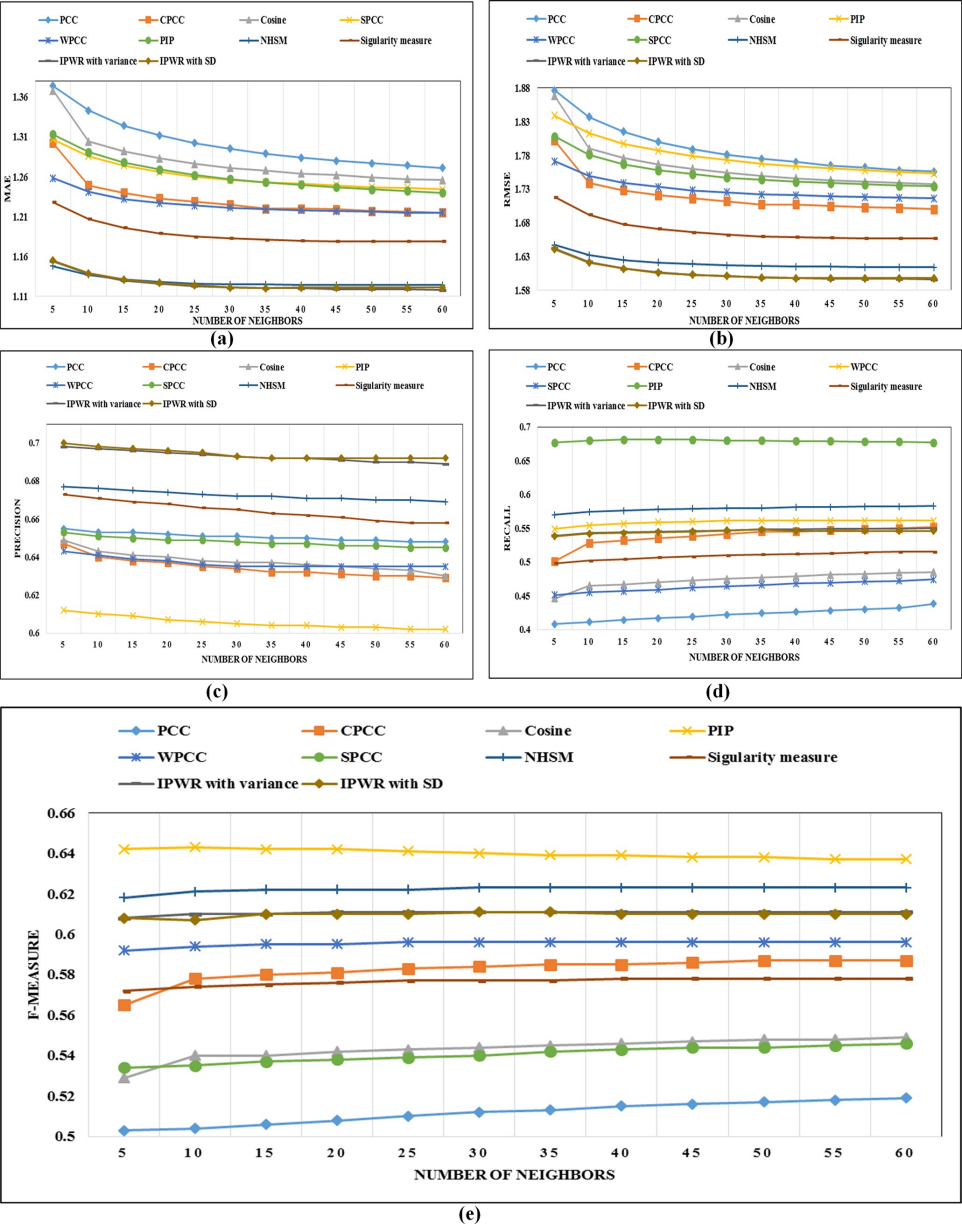
(a-e) The performance comparison of state-of-the-art similarity measure methods with the IPWR similarity measure method in terms of MAE, RMSE, precision, recall, and F-measure on the MovieTweetings dataset.

#### Performance analysis on the Epinions (5-star rating) dataset

The performance analysis of the IPWR similarity measure method with its competitor methods is presented in (Fig 3A–3E) for the Epinions dataset. (Fig 3A–3E) present performance analysis of the IPWR similarity measure method with its competitor methods in terms of the performance evaluation metrics (i.e. MAE, RMSE, Precision, Recall, and F-measure) versus the different number of neighbors. The performance analysis indicates that IPWR similarity measure method outperforms as compared to its competitor similarity measure methods in terms of the performance evaluation metrics. In order to verify the results of the IPWR similarity measure method among its competitor methods, statistical analysis is performed on the experimental results of the reported datasets using non-parametric Wilcoxon matched-pairs signed-rank test and paired t-test whose details are presented in Tables 7–10. The statistical analysis is performed by setting a standard value of the level of significance at 0.05 (95%) and results are analyzed in terms of the *z*-score, *p*-value, and *t*-score. For all the reported datasets, the value of *p* is less than the value of the significance level (i.e. *α*≤0.05), which proof the robust performance of the IPWR similarity measure method as compared to its competitor similarity measure methods. The *p*-value also indicate that the performance of the IPWR similarity measure method is strongly significant. It means that there is significant differences exist between IPWR similarity measure method and its competitor similarity measure methods. The negative sign of *z*-score also indicates the robustness of the IPWR similarity measure method as compared to its competitor similarity measure methods. Moreover, we have applied paired t-test to reassess the statistical significance of the experimental results of the IPWR similarity measure method and its competitor similarity measure methods. The value of the degrees of freedom (df) for the paired t-test is set to 11. The negative sign of *t*-score also indicates that the IPWR similarity measure method gives the best performance as compared to its competitor similarity measure methods. Furthermore, all the results of t-score are highly significant which shows that there is significant difference exist between IPWR similarity measure method and its competitor similarity measure methods.

**Table 7:** Statistical significance of the experimental results of the IPWR similarity measure method based on the MAE with state-of-the-art similarity measure methods on the Epinions dataset (* indicate the best performance).

Evaluation metrics	PCC	CPCC	Cosine	PIP	SPCC	WPCC	NHSM	Singularity measure	IPWR with variance	IPWR with SD*
**Mean error**	0.972	0.924	0.977	0.949	0.970	0.971	0.952	0.952	0.898	0.896
**Std. error**	0.0008288	0.000922	0.0011772	0.000664	0.000417	0.0004994	0.000256	0.0017238	0.0002562	0.000259
**Confidence interval**	0.9705092 - 0.9741575	0.922220 - 0.926279	0.9739924 - 0.9791743	0.947287 - 0.950212	0.968581–0.970418	0.9694842 - 0.9716824	0.951102 - 0.952230	0.9479559 - 0.9555441	0.8977694 - 0.8988973	0.895511 - 0.896655
**Statistical significance using non-parametric Wilcoxon matched-pairs signed-rank test**										
**z-score**	-3.081	-3.077	-3.077	-3.081	-3.081	-3.081	-3.126	-3.077	-3.082	NA
**p-value**	0.0021	0.0021	0.0021	0.0021	0.0021	0.0021	0.0018	0.0021	0.0021	NA
**Statistical significance using paired t-test (df-indicate degrees of freedom)**										
**Df**	11	11	11	11	11	11	11	11	11	11
**t-score**	-87.7877	-29.3989	-66.7761	-73.8322	-1.5e+02	-1.3e+02	-1.5e+02	-31.9315	-6.1648	NA
**p-value**	0.0000	0.0000	0.0000	0.0000	0.0000	0.0000	0.0000	0.0000	0.0000	NA

**Table 8:** Statistical significance of the experimental results of the IPWR similarity measure method based on the MAE with state-of-the-art similarity measure methods on the MovieLens-100K dataset (* indicate the best performance).

Evaluation metrics	PCC	CPCC	Cosine	SPCC	WPCC	PIP	NHSM	Singularity measure	IPWR with variance*	IPWR with SD
**Mean error**	0.851	0.757	0.818	0.833	0.779	0.795	0.752	0.794	0.746	0.755
**Std. error**	0.010891	0.006580	0.0100396	0.0077409	0.004230	0.0060382	0.0018	0.0059817	0.0026327	0.003584
**Confidence interval**	0.826778–0.874721	0.742350–0.771316	0.7962364 - 0.8404303	0.8157957 - 0.849871	0.770104–0.788728	0.7813767 - 0.8079566	0.7478 - 0.7561	0.7803344 - 0.8066656	0.7397888 - 0.7513779	0.747110 - 0.762889
**Statistical significance using non-parametric Wilcoxon matched-pairs signed-rank test**										
**z-score**	-3.063	-1.806	-3.068	-3.063	-3.064	-3.063	-1.927	-3.063	NA	-3.07
**p-value**	0.0022	0.0709	0.0022	0.0022	0.0022	0.0022	0.054	0.0022	NA	0.0021
**Statistical significance using paired t-test (df-indicate degrees of freedom)**										
**Df**	11	11	11	11	11	11	11	11	11	11
**t-score**	-9.3856	-1.5873	-7.0093	-10.6710	-6.7895	-7.4514	-1.9882	-7.3319	NA	-2.1173
**p-value**	0.0000	0.1267	0.0000	0.0000	0.0000	0.0000	0.0594	0.0000	NA	0.0458

**Table 9:** Statistical significance of the experimental results of the IPWR similarity measure method based on the MAE with state-of-the-art similarity measure methods on the MovieLens-1M dataset (* indicate the best performance).

Evaluation metrics	PCC	CPCC	Cosine	SPCC	WPCC	PIP	NHSM	Singularity measure	IPWR with variance*	IPWR with SD
**Mean error**	0.823	0.727	0.786	0.825	0.768	0.814	0.712	0.733	0.716	0.721
**Std. error**	0.00813	0.00327	0.00233	0.00548	0.00187	0.00855	0.00125	0.00408	0.00271	0.00271
**Confidence interval**	0.8045976 - 0.8404024	0.719540 -0.733959	0.7805382 - 0.7907951	0.812763 - 0.836903	0.763873 - 0.772126	0.794925 - 0.832575	0.70931 - 0.71485	0.7236798 - 0.741653	0.7104351 - 0.7223982	0.715182 - 0.727150
**Statistical significance using non-parametric Wilcoxon matched-pairs signed-rank test**										
**z-score**	-3.059	-3.066	-3.065	-3.061	-3.069	-3.061	1.532	-3.084	NA	-3.078
**p-value**	0.0022	0.0022	0.0022	0.0022	0.0021	0.0022	0.1255	0.002	NA	0.0021
**Statistical significance using paired t-test (df-indicate degrees of freedom)**										
**df**	11	11	11	11	11	11	11	11	11	11
**t-score**	-12.3700	-2.4279	-19.3446	-17.7141	-15.6235	-10.8457	1.4470	-3.3131	NA	-1.2357
**p-value**	0.0000	0.0238	0.0000	0.0000	0.0000	0.0000	0.1620	0.0032	NA	0.2296

**Table 10:** Statistical significance of the experimental results of the IPWR similarity measure method based on the MAE with state-of-the-art similarity measure methods on the CiaoDVD dataset (* indicate the best performance).

Evaluation metrics	PCC	CPCC	Cosine	SPCC	PIP	WPCC	NHSM	Singularity measure	IPWR with variance	IPWR with SD*
**Mean error**	0.823	0.800	0.821	0.820	0.800	0.817	0.810	0.807	0.789	0.784
**Std. error**	0.0010	0.0015625	0.001798	0.000417	0.000499	0.0004994	0.000228	0.0003658	0.0002289	0.00025
**Confidence interval**	0.8204601 - 0.8248732	0.796811 - 0.803689	0.817458–0.825374	0.818581–0.820418	0.798484 - 0.800682	0.8154842 - 0.8176824	0.809412 - 0.810420	0.8063615 - 0.8079719	0.7884128 - 0.7894205	0.7836998 - 0.7848002
**Statistical significance using non-parametric Wilcoxon matched-pairs signed-rank test**										
**z-score**	-3.129	-3.129	-3.076	-3.274	-3.274	-3.274	-3.176	-3.09	-3.176	NA
**p-value**	0.0018	0.0018	0.0021	0.0011	0.0011	0.0011	0.0015	0.002	0.0015	NA
**Statistical significance using paired t-test (df-indicate degrees of freedom)**										
**df**	11	11	11	11	11	11	11	11	11	11
**t-score**	-49.6847	-10.1116	-20.4698	-72.4471	-27.4568	-57.8981	-75.7201	-51.7188	-13.7673	NA
**p-value**	0.0000	0.0000	0.0000	0.0000	0.0000	0.0000	0.0000	0.0000	0.0000	NA

#### Performance analysis on the MovieLens-100K (ML-100K) (5-star rating) dataset

The performance comparisons in terms of the performance evaluation metrics (i.e. MAE, RMSE, Precision, Recall, and F-measure) of the IPWR similarity measure method with its competitor similarity measure methods is presented in (Fig 4A–4E) for the ML-100K dataset. After analyzing the experimental details of (Fig 4A–4E), it can be concluded that IPWR similarity measure method outperforms as compared to its competitor similarity measure methods. Furthermore, performance analysis of the IPWR similarity measure method in terms of accuracy is also better than its competitor similarity measure methods because it considers the average rating of an item and an average rating of a user simultaneously. Similarly, the RPB of a user is ignored by its competitor similarity measure methods, while IPWR similarity measure also considers user RPB, which result in improved performance.

#### Performance analysis on the MovieLens-1M (ML-1M) (5-star rating) dataset

(Fig 5A–5E) present performance analysis of the IPWR similarity measure method with its competitor similarity measure methods by a varying number of neighbors and analyzing performance in terms of the MAE, RMSE, Precision, Recall and F-measure on the ML-1M dataset. It is a large dataset with a sparsity of 95.80%. For this dataset, the IPWR similarity measure method also performs better than its competitor similarity measure methods except for the NHSM similarity measure method. The reason for the better performance of the NHSM similarity measure method is its proximity, significance, and singularity (PSS) factors, which are calculated for each common rating individually. However, the results of the NHSM similarity measure method are very close to IPWR similarity measure method. In the case of RMSE, the performance of the IPWR similarity measure method is better as compared to the NHSM similarity measure method.

#### Performance analysis on the CiaoDVD (5-star rating) dataset

(Fig 6A–6E) present performance analysis for a different number of neighbors in terms of performance evaluation metrics for the CiaoDVD dataset. In this dataset, mean ratings per user are 1.13 and mean ratings per item are 4.48. The IPWR similarity measure method also performs better on this dataset because of the consideration of the user RPB and improved PCC. It is also observed that the increase in the number of neighbors does not affect the performance of the IPWR similarity measure method. This implies that a small number of neighbors give the same results as a large number of neighbors, which also reduces the computational cost of the IPWR similarity measure method as compared to its competitor similarity measure methods.

#### Performance analysis on the MovieTweetings (10-star rating) dataset

The MovieTweetings dataset is also publicly available dataset which is crawled from twitter. This dataset consists of movie ratings in the range of [1–10]. In this rating scale, 1 indicates the worst rating and 10 indicates the best rating of a movie given by a user. This dataset contains a total of 759746 user ratings given by 56304 users using a total of 32810 movies. The detail of the user rating scales and user rating distribution for the MovieTweetings dataset is presented in Table 11. The sparsity of MovieTweetings dataset is 99.90%. The best performance of the IPWR similarity measure method is achieved by setting the weight of *α =* 0.4 and β *=* 0.6 whose experimental details are presented in Table 6 for the MovieTweetings dataset. The performance comparisons in terms of the performance evaluation metrics (i.e. MAE, RMSE, Precision, Recall, and F-measure) of the IPWR similarity measure method with its competitor similarity measure methods is presented in (Fig 7A–7E) for the MovieTweetings dataset. After analyzing the experimental details of (Fig 7A–7E), it can be concluded that IPWR similarity measure method outperforms in terms of evaluation metrics (i.e. MAE, RMSE, and Precision) as compared to its competitor similarity measure methods because it considers the average rating of an item and an average rating of a user simultaneously. Similarly, the RPB of a user is ignored by its competitor similarity measure methods, while IPWR similarity measure method also considers user RPB, which result in improved performance.

**Table 11:** User rating scale and rating distribution for the MovieTweetings dataset.

**Rating scale**	1.0	2.0	3.0	4.0	5.0	6.0	7.0	8.0	9.0	10.0
**User ratings**	9074	7945	13100	24029	56592	99,492	171593	183839	106762	87320

The statistical details of the IPWR similarity measure and its competitor similarity measure methods for the MovieTweetings dataset are presented in Table 12. The statistical analysis is performed using a non-parametric Wilcoxon matched-pairs signed-rank test and paired t-test to investigate and provide statistical evidence regarding the robust performance of the IPWR similarity measure method as compared to its competitor similarity measure methods. The value of the degrees of freedom (df) for the paired *t*-test is set to 11 for the MovieTweetings dataset. According to the statistical results presented in Table 12, the negative sign of *z*-score and *t*-score shows that IPWR similarity measure method shows the best performance as compared to its competitor methods. Furthermore, *z*-score results of the IPWR similarity measure are highly significant in all cases as its *p*-values are less than the level of significance at 0.05 (95%) however in case of *t*-score, all the results are significant except of NHSM and IPWR with SD similarity measure methods which give insignificant results due to least significant difference exist between these two methods.

**Table 12:** Statistical significance of the experimental results of the IPWR similarity measure method based on the MAE with state-of-the-art similarity measure methods on the MovieTweetings dataset (_*_ indicate the best performance).

Evaluation metrics	PCC	CPCC	Cosine	SPCC	WPCC	PIP	NHSM	Singularity measure	IPWR with variance*	IPWR with SD
**Mean error**	1.3020	1.2320	1.280	1.2615	1.2255	1.2621	1.1283	1.1887	1.1255	1.1268
**Std. error**	0.0090641	0.0070383	0.0090621	0.0054875	0.0037379	0.0064136	0.0021154	0.0043434	0.0030882	0.0030372
**Confidence interval**	1.282133 – 1.322033	1.216592 - 1.216592	1.260055 - 1.299945	1.249505 – 1.273661	1.217023 - 1.233477	1.248051 - 1.276283	1.123677 - 1.132989	1.17919 - 1.19831	1.118786 - 1.132381	1.120148 - 1.120148
**Statistical significance using non-parametric Wilcoxon matched-pairs signed-rank test**										
**z-score**	-3.059	-3.062	-3.061	-3.059	-3.061	-3.059	-2.084	-3.066	NA	-2.981
**p-value**	0.0022	0.0022	0.0022	0.0022	0.0022	0.0022	0.0372	0.0022	NA	0.0029
**Statistical significance using paired t-test (df-indicate degrees of freedom)**										
**df**	11	11	11	11	11	11	11	11	11	11
**t-score**	-18.4319	-13.8564	-16.129	-21.5981	-20.5558	-19.1875	-0.7347	-11.8524	NA	-0.2886
**p-value**	0.0000	0.0000	0.0000	0.0000	0.0000	0.0000	0.4703	0.0000	NA	0.7756

## Conclusion and future work

In this article, we identify and analyze some limitations of the state-of-the-art similarity measure methods, especially the PCC similarity measure method. These similarity measures are used by collaborative filtering based methods to find similar users’ and items’ profiles. User RPB is one of the most important aspects, which is ignored by traditional similarity measurement methods. Typically, different users have different RPB and based upon this behavior, they tend to rate items with values that have not many variations. In this article, we have proposed an improved similarity measure method that uses the user’s RPB pattern to find similar users. The RPB pattern is modeled as a function of user rating averages and user variance or standard deviation. The proposed IPWR similarity measure method overcomes some inherent shortcomings of a standard PCC similarity measure and it also considers the RPB pattern of users to achieve better performance. The extensive experiments are performed to check the effectiveness of the IPWR similarity measure method. The performance of the IPWR similarity measure method is compared against state-of-the-art similarity measure methods using four publically available datasets. The results show that the IPWR similarity measure performs better than conventional and state-of-the-art similarity measure methods like NHSM and PIP. It is also observed from experimental results that IPWR similarity measure method performs better on sparse datasets (i.e. Epinions, CiaoDVD, and MovieTweetings datasets) than dense datasets (i.e. ML-100K and ML-1M datasets). In future work, we intend to learn weights of α and β using various machine learning methods such as support vector machine (SVM), particle swarm intelligence (PSO), and artificial neural networks (ANN). Although the IPWR similarity measure method considers both local and global information of user ratings in terms of user RPB, one important information, which is the actual rating value of non-co-rated items are ignored. In the future, we will also try to incorporate this information using the IPWR similarity measure method. Furthermore, a friendship network of a user can also be used as an additional information source in the extremely cold start or sparse conditions.

## References

[1] Boratto L., Carta S., and Fenu G., Investigating the role of the rating prediction task in granularity-based group recommender systems and big data scenarios. Information Sciences, 2017 378: p. 424–443.

[2] Prugel-Bennett, M.A.G.a.A., *A Scalable, Accurate Hybrid Recommender System*. IEEE Third International Conference on Knowledge Discovery and Data Mining, 2010: p. 94–98.

[3] Li Y.-M., Wu C.-T., and Lai C.-Y., A social recommender mechanism for e-commerce: Combining similarity, trust, and relationship. Decision Support Systems, 2013 55(3): p. 740–752.

[4] https://www.amazon.com/, Accessed on 14th May 2018.

[5] Shumeet B., Rohan S., Sivakumar D., Jing Y., Yagnik J., Kumar S., et al., *Video suggestion and discovery for YouTube: taking random walks through the view graph*. International Conference on World Wide Web, 2008: p. 895–904.

[6] https://news.google.com/, Accessed on 14th May 2018.

[7] Ghazanfar, M.A. and Prugel-Bennett, A. *A scalable, accurate hybrid recommender system. in 2010 Third International Conference on Knowledge Discovery and Data Mining*. 2010. IEEE.

[8] Ayub, M., Ghazanfar, M.A., Maqsood, M., and Saleem, A. *A Jaccard base similarity measure to improve performance of CF based recommender systems* in *32nd International Conference on Information Networking (ICOIN)*. 2018. IEEE.

[9] Zahra S., Ghazanfar M.A., Khalid A., Azam M.A., Naeem U., and Prugel-Bennett A., Novel centroid selection approaches for KMeans-clustering based recommender systems. Information sciences, 2015 320: p. 156–189.

[10] Xiong, L., Chen, X., Huang, T.-K., Schneider, J., and Carbonell, J.G. *Temporal collaborative filtering with bayesian probabilistic tensor factorization* in *Proceedings of the 2010 SIAM international conference on data mining*. 2010. SIAM.

[11] Ghazanfar, M.A. and Prϋgel-Bennett, A. *Exploiting context in kernel-mapping recommender system algorithms* in *Sixth International Conference on Machine Vision (ICMV 2013)*. 2013. International Society for Optics and Photonics.

[12] Adomavicius G. and Tuzhilin A., Toward the next generation of recommender systems: A survey of the state-of-the-art and possible extensions. IEEE Transactions on Knowledge & Data Engineering, 2005(6): p. 734–749.

[13] Bobadilla J., Ortega F., Hernando A., and Gutiérrez A., Recommender systems survey. Knowledge-based systems, 2013 46: p. 109–132.

[14] Ricci F., Rokach L., and Shapira B., *Introduction to recommender systems handbook*. 2011: Springer.

[15] Mehmood Z., Anwar S.M., Ali N., Habib H.A., and Rashid M., A novel image retrieval based on a combination of local and global histograms of visual words. Mathematical Problems in Engineering, 2016 2016.

[16] Mehmood Z., Anwar S.M., and Altaf M., A novel image retrieval based on rectangular spatial histograms of visual words. Kuwait Journal of Science, 2018 45(1).

[17] Mehmood Z., Abbas F., Mahmood T., Javid M.A., Rehman A., and Nawaz T., Content-based image retrieval based on visual words fusion versus features fusion of local and global features. Arabian Journal for Science and Engineering, 2018 43(12): p. 7265–7284.

[18] Alahmadi, D.H. and Zeng, X.-J. *Twitter-based recommender system to address cold-start: A genetic algorithm based trust modelling and probabilistic sentiment analysis* in *2015 IEEE 27th International Conference on Tools with Artificial Intelligence (ICTAI)*. 2015. IEEE.

[19] Lee W.-P. and Ma C.-Y., Enhancing collaborative recommendation performance by combining user preference and trust-distrust propagation in social networks. Knowledge-Based Systems, 2016 106: p. 125–134.

[20] Ahn H.J., A new similarity measure for collaborative filtering to alleviate the new user cold-starting problem. Information Sciences, 2008 178(1): p. 37–51.

[21] Guo, G., *Integrating trust and similarity to ameliorate the data sparsity and cold start for recommender systems*. Proceedings of ACM Conference on Recommender Systems, 2013: p. 451–454.

[22] Safoury L. and Salah A., Exploiting user demographic attributes for solving cold-start problem in recommender system. Lecture Notes on Software Engineering, 2013 1(3): p. 303–307.

[23] Guo G., Jie Zhang, and Daniel Thalmann., Merging trust in collaborative filtering to alleviate data sparsity and cold start. Knowledge-Based Systems, 2014 57: p. 57–68.

[24] Al-Oufi S., Kim H.-N., and El Saddik A., A group trust metric for identifying people of trust in online social networks. Expert Systems with Applications, 2012 39(18): p. 13173–13181.

[25] Golbeck, J. and Hendler, J. *Filmtrust: Movie recommendations using trust in web-based social networks* in *Proceedings of the IEEE Consumer communications and networking conference*. 2006. Citeseer.

[26] Liu, B.Y.Y.L.D. and Liu, J. *Social Collaborative Filtering by Trust* in *Proceedings of the Twenty-Third International Joint Conference on Artificial Intelligence, IEEE*. Citeseer.

[27] Bobadilla J., F.O., Hernando A., Bernal J., A collaborative filtering approach to mitigate the new user cold start problem. Knowledge-Based Systems, 2011 26: p. 225–238.

[28] Bobadilla J., F.O., Hernando A., Bernal J., A collaborative filtering similarity measure based on singularities, *Inform*. *Process*. *Manage*. 2012 48: p. 204–217.

[29] Jabeen S., Mehmood Z., Mahmood T., Saba T., Rehman A., and Mahmood M.T., An effective content-based image retrieval technique for image visuals representation based on the bag-of-visual-words model. PloS one, 2018 13(4): p. e0194526 10.1371/journal.pone.0194526 29694429 PMC5919049

[30] Sarwar A., Mehmood Z., Saba T., Qazi K.A., Adnan A., and Jamal H., A novel method for content-based image retrieval to improve the effectiveness of the bag-of-words model using a support vector machine. Journal of Information Science, 2019 45(1): p. 117–135.

[31] Mehmood Z., Rashid M., Rehman A., Saba T., Dawood H., and Dawood H., Effect of complementary visual words versus complementary features on clustering for effective content-based image search. Journal of Intelligent & Fuzzy Systems, 2018(Preprint): p. 1–14.

[32] Qazi K.A., Nawaz T., Mehmood Z., Rashid M., and Habib H.A., A hybrid technique for speech segregation and classification using a sophisticated deep neural network. PloS one, 2018 13(3): p. e0194151 10.1371/journal.pone.0194151 29558485 PMC5860734

[33] Liu H., Hu Z., Mian A., Tian H., and Zhu X., A new user similarity model to improve the accuracy of collaborative filtering. Knowledge-Based Systems, 2014 56: p. 156–166.

[34] Jamali, M. and Ester, M. *Using a trust network to improve top-N recommendation* in *Proceedings of the third ACM conference on Recommender systems*. 2009. ACM.

[35] Bobadilla J., Ortega F., and Hernando A., A collaborative filtering similarity measure based on singularities. Information Processing & Management, 2012 48(2): p. 204–217.

[36] Bobadilla J., Hernando A., Ortega F., and Gutiérrez A., Collaborative filtering based on significances. Information Sciences, 2012 185(1): p. 1–17.

[37] Ma H., King I., and Lyu M.R., Learning to recommend with explicit and implicit social relations. ACM Transactions on Intelligent Systems and Technology (TIST), 2011 2(3): p. 29.

[38] Gong, S., Ye, H., and Dai, Y. *Combining singular value decomposition and item-based recommender in collaborative filtering* in *2009 Second International Workshop on Knowledge Discovery and Data Mining*. 2009. IEEE.

[39] Szwabe, A., Ciesielczyk, M., and au>Janasiewicz, T. *Semantically enhanced collaborative filtering based on RSVD* in *International Conference on Computational Collective Intelligence*. 2011. Springer.

[40] Polatidis N. and Georgiadis C.K., A multi-level collaborative filtering method that improves recommendations. Expert Systems with Applications, 2016 48: p. 100–110.

[41] Patra B.K., Launonen R., Ollikainen V., and Nandi S., A new similarity measure using Bhattacharyya coefficient for collaborative filtering in sparse data. Knowledge-Based Systems, 2015 82: p. 163–177.

[42] Sun S.-B., Zhang Z.-H., Dong X.-L., Zhang H.-R., Li T.-J., Zhang L., et al, Integrating Triangle and Jaccard similarities for recommendation. PloS one, 2017 12(8): p. e0183570 10.1371/journal.pone.0183570 28817692 PMC5560696

[43] Saranya K. and Sadasivam G.S., Modified heuristic similarity measure for personalization using collaborative filtering technique. Applied Mathematics and Information Sciences, 2017 11(1): p. 317–25.

[44] Ayub, M., Ghazanfar, M.A., Maqsood, M., and Saleem, A. *A Jaccard base similarity measure to improve performance of CF based recommender systems* in *2018 International Conference on Information Networking (ICOIN)*. 2018. IEEE.

[45] Tan Z. and He L., An efficient similarity measure for user-based collaborative filtering recommender systems inspired by the physical resonance principle. IEEE Access, 2017 5: p. 27211–27228.

[46] http://www.trustlet.org/downloaded_epinions.html.

[47] http://www.movielens.umn.edu.

[48] Harper F.M. and Konstan J.A., The movielens datasets: History and context. Acm transactions on interactive intelligent systems (tiis), 2016 5(4): p. 19.

[49] Guo, G., Zhang, J., Thalmann, D., and Yorke-Smith, N. *Etaf: An extended trust antecedents framework for trust prediction* in *Proceedings of the 2014 IEEE/ACM International Conference on Advances in Social Networks Analysis and Mining*. 2014. IEEE Press.

[50] https://github.com/sidooms/MovieTweetings/tree/master/latest.

